# Hydraulic differences between flowers and leaves are driven primarily by pressure-volume traits and water loss

**DOI:** 10.3389/fpls.2023.1130724

**Published:** 2023-05-31

**Authors:** Yi-Dong An, Adam B. Roddy, Tian-Hao Zhang, Guo-Feng Jiang

**Affiliations:** ^1^ Guangxi Key Laboratory of Forest Ecology and Conservation, Guangxi Colleges and Universities Key Laboratory for Cultivation and Utilization of Subtropical Forest Plantation, and State Key Laboratory for Conservation and Utilization of Subtropical Agro-Bioresources, College of Forestry, Guangxi University, Nanning, Guangxi, China; ^2^ Institute of Environment, Department of Biological Sciences, Florida International University, Miami, FL, United States

**Keywords:** hydraulics, water relations, xylem, flower, drought tolerance, minimum cuticular conductance, photosynthesis, leaf

## Abstract

Flowers are critical for successful reproduction and have been a major axis of diversification among angiosperms. As the frequency and severity of droughts are increasing globally, maintaining water balance of flowers is crucial for food security and other ecosystem services that rely on flowering. Yet remarkably little is known about the hydraulic strategies of flowers. We characterized hydraulic strategies of leaves and flowers of ten species by combining anatomical observations using light and scanning electron microscopy with measurements of hydraulic physiology (minimum diffusive conductance (*g*_min_) and pressure-volume (PV) curves parameters). We predicted that flowers would exhibit higher *g*_min_ and higher hydraulic capacitance than leaves, which would be associated with differences in intervessel pit traits because of their different hydraulic strategies. We found that, compared to leaves, flowers exhibited: 1) higher *g*_min_, which was associated with higher hydraulic capacitance (*C*_T_); 2) lower variation in intervessel pit traits and differences in pit membrane area and pit aperture shape; and 3) independent coordination between intervessel pit traits and other anatomical and physiological traits; 4) independent evolution of most traits in flowers and leaves, resulting in 5) large differences in the regions of multivariate trait space occupied by flowers and leaves. Furthermore, across organs intervessel pit trait variation was orthogonal to variation in other anatomical and physiological traits, suggesting that pit traits represent an independent axis of variation that have as yet been unquantified in flowers. These results suggest that flowers, employ a drought-avoidant strategy of maintaining high capacitance that compensates for their higher *g*_min_ to prevent excessive declines in water potentials. This drought-avoidant strategy may have relaxed selection on intervessel pit traits and allowed them to vary independently from other anatomical and physiological traits. Furthermore, the independent evolution of floral and foliar anatomical and physiological traits highlights their modular development despite being borne from the same apical meristem.

## 1 Introduction

Flowers play a crucial role during the reproductive phase of angiosperms, and their importance during this period has influenced angiosperm diversification and spread (Crane et al., 1995; Sprengel, 1996; Sargent and Ackerly, 2008). Producing and maintaining flowers requires the allocation of resources, such as water, carbon, and nutrients (Bazzaz et al., 1987; Reekie and Bazzaz, 1987a; Reekie and Bazzaz, 1987b; Reekie and Bazzaz, 1987c). Though these physiological costs of flowers are often assumed to be minimal in the context of the whole plant, resource limitation or stressful abiotic conditions can exacerbate the costs of producing and maintaining flowers (Lambrecht, 2013; Burkle and Runyon, 2016; Waser and Price, 2016; Bourbia et al., 2020; Harrison Day et al., 2022). The abiotic conditions that influence the physiological costs also act as agents of selection on floral traits and, in general, can be as strong an agent of selection on flowers as pollinators (Ashman and Schoen, 1994; Galen et al., 1999; Caruso, 2006; Lambrecht and Dawson, 2007; Teixido and Valladares, 2014; Lambrecht et al., 2017; Roddy et al., 2018; Caruso et al., 2019; Roddy et al., 2019; Kuppler and Kotowska, 2021a; Kuppler et al., 2021b). One of the most important resources for plant growth and reproduction is water, and the frequency and severity of droughts is increasing globally (Adams et al., 2017; Choat et al., 2018; Brodribb et al., 2020). These droughts potentially threaten food production and other ecosystem services that rely on flowering. Thus, maintaining water balance is critical to flower functioning, successful reproduction, and flower and fruit development (Galen et al., 1999; Lambrecht, 2013; Roddy et al., 2016; Zhang et al., 2018; Bourbia et al., 2020).

Like leaves, flowers are terminal structures often located in the hottest and driest parts of the plant crown, meaning they are exposed to similar evaporative environments as leaves (Blanke and Lovatt, 1993; Roddy and Dawson, 2012). In order to optimize photosynthesis in leaves, plants must prevent declines in water content, which requires coordination in the structural traits governing water flow through each component of the plant hydraulic pathway (Brodersen et al., 2014; Jupa et al., 2017; Li et al., 2019; Song et al., 2021; Fontes et al., 2022). For example, coordination between leaf vein density and stomatal density is nearly ubiquitous across studies and highlights the important roles that leaf veins and stomata play in coordinating liquid and vapor fluxes through the leaf to maintain water balance (Sack and Frole, 2006; Brodribb et al., 2007; Noblin et al., 2008; Boyce et al., 2009; Brodribb and Cochard, 2009; de Boer et al., 2012). However, over short timescales, water loss can exceed water supply, causing declines in water potentials in the xylem. Under extreme cases, excessive water loss and low water potentials can pull air into the xylem vessels from either outside the xylem or from adjacent, already embolized vessels, leading to the spread of air embolisms and xylem dysfunction (Dixon and Joly, 1895; Sperry and Tyree, 1988; Lens et al., 2011; Tyree and Zimmermann, 2013).

One of the major determinants of both the vulnerability of the xylem to embolism spread and also the efficiency of water flow through the xylem is the structure of intervessel pits and pit membranes that connect adjacent xylem conduits. Pit membranes, in particular, can be responsible for 50% or more of the total hydraulic resistance in the xylem (Wheeler et al., 2005; Choat et al., 2006; Hacke et al., 2006; Pittermann et al., 2006; Kaack et al., 2019). Comparative studies have shown that pit morphology can vary in terms of pit and pit aperture size, pit shape, and pit density, and that these pit traits can correlate with vessel diameter, vessel wall thickness, and photosynthetic rates, and vary both among species and among habitats (Schmitz et al., 2007; Lens et al., 2011; Jacobsen et al., 2016; Li et al., 2019; Zhang et al., 2021). In general, larger pit membranes are associated with higher hydraulic conductivity but are more vulnerable to embolism (Choat et al., 2005; Wheeler et al., 2005; Ellmore et al., 2006; Hacke et al., 2006; Lens et al., 2011). Furthermore, pit aperture shape can also influence embolism resistance: species with more cavitation-resistant branches exhibit narrower and more elliptical pit apertures (Lens et al., 2011; Scholz et al., 2013; Li et al., 2019). Thus, intervessel pit traits are important factors influencing both hydraulic safety and efficiency (Hacke et al., 2009; Jansen et al., 2009; Blackman et al., 2010; Li et al., 2016; Zhang et al., 2021), though they have not been systematically quantified in reproductive organs.

Compared to leaves, relatively little is known about the hydraulic traits of flowers, despite their importance to reproduction for most species (Gleason, 2018). Flowers have, at most, very few stomata (Lipayeva, 1989; Roddy et al., 2016; Zhang et al., 2018), meaning that water loss occurs primarily *via* diffusion across the cuticle (Roddy, 2019), and very low vein densities compared to leaves (Roddy et al., 2013; Zhang et al., 2018). As a result, minimum diffusive conductance (*g*_min_) (Kerstiens, 1996) is strongly coordinated with petal vein density and hydraulic conductance, suggesting that *g*_min_ is critical to floral water balance and hydraulic conductance (Roddy et al., 2016). This has important implications for water balance during drought conditions. While leaves can close their stomata to limit water loss (Meinzer, 2002), without stomata flowers are likely unable to curtail water loss (Roddy et al., 2016; Roddy, 2019), which can cause them to lose more water during drought than leaves (Lambrecht, 2013; Bourbia et al., 2020). Thus, flower water potentials may decline more quickly than leaf water potentials and possibly cause air embolisms to spread more quickly through the xylem in flowers than in leaves (Zhang and Brodribb, 2017; Bourbia et al., 2020), depending on the morphology of intervessel pit traits in flowers and leaves (Zhang et al., 2021). However, hydraulic capacitance can buffer declines in water potentials that lead to embolism spread, and flowers have significantly higher hydraulic capacitance than leaves (Roddy et al., 2019). Since flowers are short-lived but have high water demands (Roddy and Dawson, 2012; Lambrecht, 2013; Roddy et al., 2018; Bourbia et al., 2020), flowers might employ different hydraulic strategies than leaves and stems and exhibit different coordination between hydraulic traits than leaves. High water demands and greater reliance on stored water may physiologically buffer flowers from diurnal variability in the water status of other plant structures. Prior evidence based on 132 species has suggested that vegetative and reproductive structures may be developmentally modular, with independent evolution of vein density in flowers and leaves (Roddy et al., 2013). Similarly, based on data from about 20 species, flowers tend to have higher water contents and hydraulic capacitance than leaves. These differences in venation and pressure-volume traits may be linked to other differences in hydraulic anatomy and physiology. Yet remarkably little is known about the hydraulic strategies of flowers and their mechanisms of maintaining water balance.

In the present study, we characterized a diverse set of anatomical and physiological traits in both leaves and flowers of ten angiosperm species (**Tables 1**, **2**). These traits included vein and stomatal traits, minimum diffusive conductance (*g*_min_), parameters derived from pressure-volume curves (Scholander et al., 1965; Tyree and Hammel, 1972), and pit traits measured using scanning electron microscopy (SEM). We used this diverse set of traits to address the following questions (1) Do species with higher *g*_min_ have higher hydraulic capacitance, which could buffer water potential declines due to excessively high *g*_min_? (2) Do flowers and leaves exhibit differences in intervessel pit structure reflecting their different hydraulic strategies? (3) Are anatomical and physiological traits in leaves and flowers coordinated, which would indicate similar hydraulic strategies? We hypothesized that flowers would exhibit higher *g*_min_ and higher hydraulic capacitance than leaves. Since flowers may rely on high water content and hydraulic capacitance to support high *g*_min_, we also hypothesized that intervessel pit traits and the coordination of anatomical and physiological traits would differ in flowers and leaves and indicate different hydraulic strategies.

**Table 1:** List of species in this study.

Species	Family	Genus	Code
*Bauhinia* × *blakeana* Dunn	Fabaceae	*Bauhinia*	*Bb*
*Bidens pilosa* L.	Asteraceae	*Bidens*	*Bp*
*Bougainvillea spectabilis* Willd.	Nyctaginaceae	*Bougainvillea*	*Bs*
*Catharanthus roseus* (L.) G. Don	Apocynaceae	*Catharanthus*	*Cr*
*Ceiba speciosa* (A.St.-Hil.) Ravenna	Malvaceae	*Ceiba*	*Cs*
*Hibiscus rosa-sinensis* L.var. *rubro-plenus* Sweet	Malvaceae	*Hibiscus*	*Hr*
*Michelia* × *alba* DC.	Magnoliaceae	*Michelia*	*Ma*
*Rhododendron* sp.	Ericaceae	*Rhododendron*	*Rh*
*Rosa* sp.	Rosaceae	*Rosa*	*Ro*
*Ruellia simplex* C.Wright	Acanthaceae	*Ruellia*	*Rs*

**Table 2:** List of major characters with definition and units.

Symbol	Definition		Units
Minimum diffusive conductance and theoretical hydraulic conductivity			
*g*_min,area_	Minimum diffusive conductance (normalized by the projected area of each organ)		mmol m^-2^ s^-1^
*g*_min,mass_	Minimum diffusive conductance (normalized by the dry mass of each organ)		mmol g^-1^ s^-1^
*K*_th_	Theoretical hydraulic conductivity		kg m^-1^ MPa^-1^ s^-1^
Pressure–volume parameters			
*C*_T_	Absolute capacitance, normalized by dry mass		mol kgs^-1^ MPa^-1^
SWC	Saturated water content		g g^-1^
Ψ_sft_	Osmotic potential at full turgor		MPa
Ψ_tlp_	Water potential at turgor loss point		MPa
SEM anatomical traits			
*D*_pml_	Diameter of the outer pit membrane along the longest axis		µm
*D*_pms_	Diameter of the outer pit membrane along the shortest axis		µm
*D*_pal_	Diameter of the outer pit aperture along the longest axis		µm
*D*_pas_	Diameter of the outer pit aperture along the shortest axis		µm
*A*_pit_	Intervessel pit membrane surface area		µm^2^
*A*_pa_	Intervessel pit aperture surface area		µm^2^
*R*_pa_	Pit aperture shape = ratio of the longest axis of outer pit aperture to the shortest axis		
*R*_pit_	Pit membrane shape = ratio of longest axis of outer pit membrane to the shortest axis		
*D*_p_	Pit density = number of intervessel pits per vessel wall area		no.µm^-2^
LM anatomical traits			
*S*_s_	Stomatal size		µm^2^
*D*_s_	Stomatal density		no.mm^-2^
*D*_v_	Vein density		mm mm^-2^
LT	Leaf thickness		µm
FT	Flower thickness		µm
*D*_h_	Hydraulically-weighted vessel diameter		µm
*T*_w_	Double vessel wall thickness		µm
VF	Vessel frequency		no.mm^-2^

## 2 Materials and methods

### 2.1 Plant species and study site

**Figure 1:**
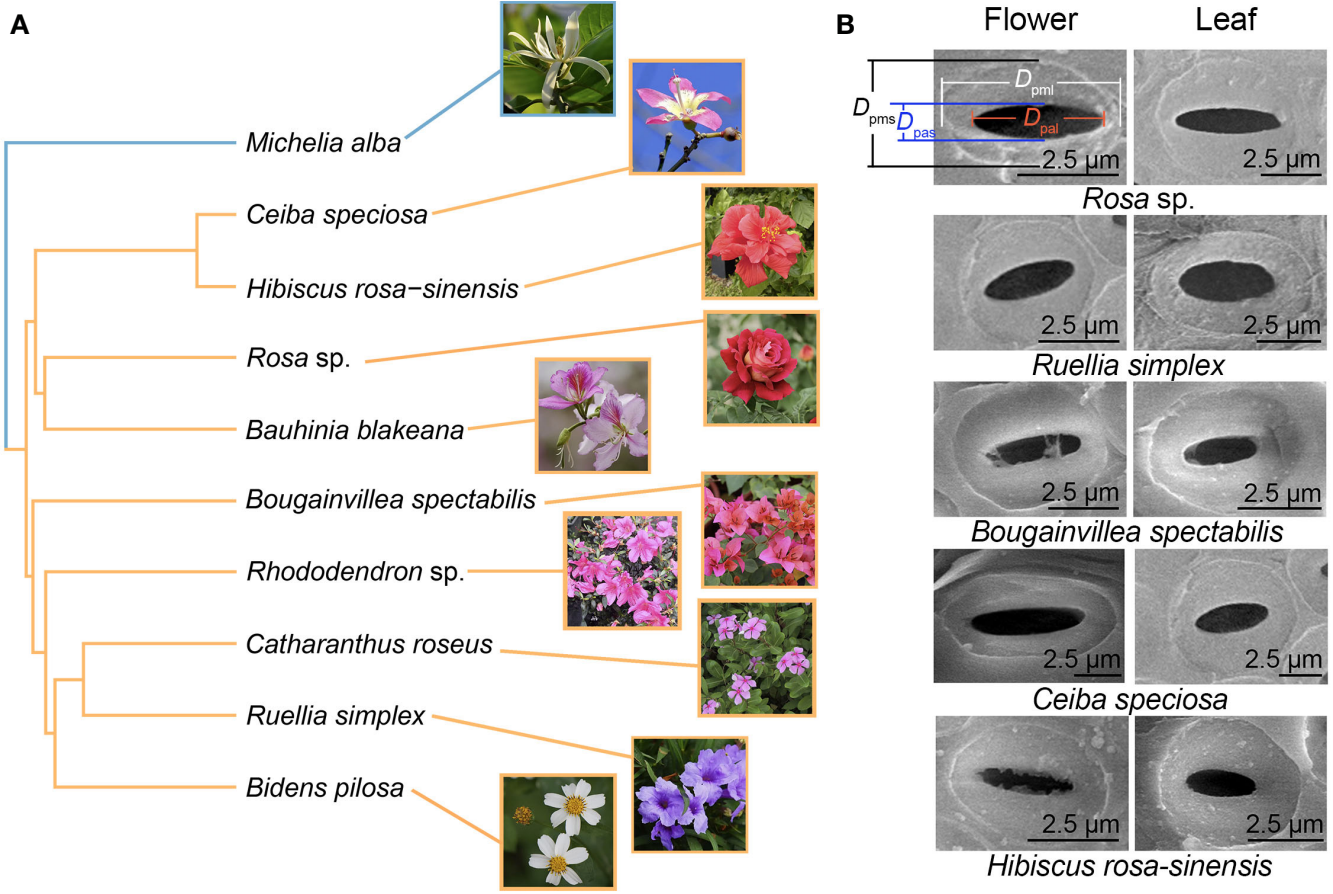
Phylogenetic relationships of the 10 species **(A)** and scanning electron micrographs of intervessel pits **(B)**. The bule and yellow branches represent magnoliids and eudicots, respectively.

Flower and leaf samples of the 10 species in this study (**Figure 1** and **Table 1**) were collected on the campus of Guangxi University, Nanning (Guangxi, China, 22°50′N 108°17′E), which has a subtropical monsoon climate with a mean annual temperature of 21.8°C and a mean annual precipitation of 1,290 mm. Three to five randomly selected individuals per species were selected for sampling. On each plant, a sun-exposed branch with leaves and flowers was cut and immediately placed in a bucket with water in the evening or early morning and transported back to the laboratory on campus.

### 2.2 Light microscopy of anatomical traits

All measurements were made on a fully expanded, healthy, sun-exposed branch with flowers and leaves for each of the 3-5 individuals sampled per species. From each leaf or flower, approximately 1-cm^2^ sections of lamina were excised, avoiding the margin and midrib. These sections were cleared in a 1:1 solution of H_2_O_2_ (30%) and CH_3_COOH (100%), then incubated at 70°C until all pigments had been removed. Sections were then removed from this solution and rinsed in water for 3 minutes, then the epidermises separated with forceps from the mesophyll and veins, allowing the upper and lower epidermises to be stained and mounted separately. To increase contrast, all samples were stained with Safranin O (0.5% w/v in water) for 5 min and Alcian Blue (1% w/v in 3% acetic acid) for 20 secs - 1 min, then washed in water and mounted on microscope slides.

Cross-sections of petals and leaves were made with a sliding microtome (RM225, Leica Inc., Germany) with a tissue thickness of 35 µm. Cross-sections with the same thickness were also made of peduncles and petioles. Sections were bleached for 10 min, rinsed in water, and then stained with Safranin O (0.5% w/v in water) for 5 min and with Alcian Blue (1% w/v in 3% acetic acid) for 20 secs - 1 min, rinsed, and then mounted on glass slides.

Images were taken at 5x, 10x, or 20x magnification, which had fields of view of approximately 3.99 mm^2^, 0.89 mm^2^, and 0.22 mm^2^, respectively, using a compound microscope outfitted with a digital camera (DM3000, Leica Inc., Germany). Both abaxial (lower) and adaxial (upper) leaf and petal surfaces were imaged for all species to determine whether they were amphistomatous. In subsequent analyses, we used sum of abaxial and adaxial stomatal densities for comparisons. We found no stomata on petals of *Catharanthus roseus* and *Rosa* sp.

All anatomical measurements from images were made using ImageJ (Rueden et al., 2017). From images of paradermal sections, vein density (*D*_v_) was measured as the total length of leaf or petal vascular tissue per mm^2^ of leaf area or petal area, stomatal density (*D*_s_) was measured by counting the number of stomata in the image and dividing by the area of the field of view, stomatal size (*S*_s_) (comprising a pair of guard cells) was directly measured on at least five stomata per image. Partial stomata and epidermal cells were included in the density counts if visible along the top and left borders of the photomicrographs and discarded if visible along the bottom and right borders (Carins Murphy et al., 2017). Leaf and petal thicknesses were directly measured from the cross-section images.

Vessel double wall thickness (*T*_w_) was measured on at least 10 pairs of connected vessels per image from cross-sections of peduncles and petioles. Mean hydraulically weighted vessel diameter (*D*_h_) for each species was calculated as (Tyree and Zimmermann, 2013):


$$D_{h}={\left[\frac{\sum^{}D^{4}}{N}\right]}^{0.25}$$

where *D* is the equivalent circular diameter of a vessel whose area was calculated from its long and short diameters and *N* is the number of vessels measured. The *D*_h_ is biased towards wider vessels that conduct the majority of water according to the Hagen–Poiseuille law. Average vessel frequency (VF) was calculated per image by dividing the total number of vessels by the image area. For each species, we calculated the theoretical hydraulic conductivity of as (Rakthai et al., 2020):


$$K_{th}=\frac{\;\pi \rho }{128 \eta }\times VF\times {Dh}_{{\;}^{4}}$$

where *ρ* is fluid density (assumed to be 998.2 kg m^-3^ at 20°C) and η is viscosity of water (1.002 x 10^–9^ MPa s^-1^ at 20°C).

### 2.3 Scanning electron microscopy of anatomical traits

Upon returning samples to the lab, peduncles and petioles were immediately cut into small segments and placed in 100 ml of 5% FAA fixative (90:5:5 ratio of 70% ethanol, acetic acid, formaldehyde) at room temperature (25°C) to prevent expansion or shrinkage. Longitudinal sections of the segments were made with a sliding microtome (RM225, Leica Inc., Germany) at a thickness of 2-3 mm. The sections were fixed to aluminum sample holders with an electron-conductive carbon adhesive tape (Nisshin EM Co. Ltd., Tokyo), air-dried for 12 h at room temperature, and coated with gold using a sputter coater (Cressington 108Auto) for 40 secs at 0.08 mA to get a 20-nm-thick gold layer, under an argon atmosphere. A conventional scanning electron microscope (FEI Quattro S, US) with a voltage of 2 kV was used to visualize intervessel pit parameters according to standard protocols (Jansen et al., 2009; Lens et al., 2011; Zhang et al., 2021).

ImageJ (Rueden et al., 2017) was used to determine the following intervessel pit characteristics (**Table 2**): intervessel pit aperture surface area (*A*_pa_), intervessel pit surface area or intervessel pit membrane surface area (*A*_pit_), pit aperture longest diameter (*D*_pal_), pit aperture shortest diameter (*D*_pas_), pit aperture shape (*R*_pa_ = *D*_pal_/*D*_pas_), pit membrane longest diameter (*D*_pml_), pit membrane shortest diameter (*D*_pms_), pit shape or pit membrane shape (*R*_pit_ = *D*_pml_/*D*_pms_) and pit density (*D*_p_). Mean values of these intervessel pit traits were calculated from at least 50 measurements from SEM images of various intervessel walls per individual.

### 2.4 Measurement of pressure–volume parameters

Shoots with leaves and flowers were collected from at least three individuals per species at night or at predawn and transported back to the laboratory. In the lab, all shoots were recut underwater to rehydrate for at least 2 h and covered with a black plastic bag during equilibration. Initial water potentials were checked and always close to -0.1 MPa. Pressure–volume curves were constructed for each sample by repeatedly measuring the bulk water potential using a pressure chamber (0.01 MPa resolution; PMS Instruments, Albany, OR, USA) and the mass to determine the relationship between water potential and water content following standard methods (Scholander et al., 1965; Tyree and Hammel, 1972; Sack and Pasquet-Kok, 2011; Roddy et al., 2019; Jiang et al., 2022). Prior to each water potential measurement, samples were enclosed in humidified plastic bags for about 20 min to allow equilibration. The pressure chamber was kept humidified with wet paper towels to prevent evaporation during the water potential measurement. After water potential measurement, the sample was weighed on a balance (± 0.0001g, model ML204T; Mettler Toledo). At the end of measurements, samples were oven-dried at 70°C for at least 72 h before determining dry mass. Because measuring flower surface area is difficult after turgor loss, pressure–volume parameters were expressed on a dry mass basis, according to previous analyses (Roddy et al., 2019). From these pressure-violume curves, we calculated saturated water content (SWC), absolute capacitance (C_T_), water potential at turgor loss point (Ψ_tlp_), and osmotic potential at full turgor (Ψ_sft_) (**Table 2**).

### 2.5 Leaf and flower minimum diffusive conductance

Shoots with leaves and flowers were collected at night from at least five individuals per species, recut underwater, and rehydrated over night while covered with a black plastic bag. Leaf and flower samples were excised in the morning, including the petiole or peduncle. Immediately following excision, their cut ends were sealed with glue and the entire organ was weighed every 10 min using an electronic balance ( ± 0.0001g, model ML204T; Mettler Toledo) in a dark room. The room was equipped with an air-conditioning to control the temperature and humidity, and samples were hung in front of a large fan as they desiccated. The velocity of air flow was high enough to physically move the samples. A small temperature and humidity sensor was kept near the samples, and temperature (T) and relative humidity (RH) were recorded manually each time a sample was weighed. After ten measurements, samples were scanned to determine projected area and then oven-dried at 70°C for 72 hours before determining dry mass.

Minimum diffusive conductance (*g*_min_) was calculated as (Bourbia et al., 2020):


$$g_{min}=\frac{WL\cdot P_{atm}}{VPD}$$

where WL is the water loss rate (mmol m^-2^ s^-1^) calculated as the slope of mass (g) over time (s) and normalized by the projected area (m^2^) or dry mass (g) of each organ; P_atm_ is the atmospheric pressure (101.3 kPa); VPD is the vapor pressure deficit determined using the Arden Buck equation (Buck, 1981).


$$VPD=\left(1-\frac{RH}{100}\right)\left(0.61121\times e^{\frac{17.502T}{240.97+T}}\right)$$

### 2.6 Data analysis

All statistical analyses were conducted in R (v. 4.0.3) (Core Team, 2022). Paired *t*-tests were used to determine differences between flowers and leaves. Differences of pit area and density among different plant lineages were tested through one-way ANOVA. We used linear regression and standard major axis (SMA) regression (R package ‘smatr’) to determine the relationships between traits (Warton et al., 2012). Principal component analysis (PCA) was carried out on centered and scaled trait data using the ‘vegan’ package. A phylogenetic tree was built using the R package ‘V.PhyloMaker’ and phylogenetic independent contrasts (PICs) were calculated using the ‘pic’ function in the R package ‘ape’ and PIC correlations tested using linear regression. All statistical tests were considered significant at *P*< 0.05. In order to contextualize our measurements of inter-conduit pit traits on leaves and flowers, we compiled published data reporting pit membrane surface area (*A*_pit_) and pit density (*D*_p_) for a diverse set of vascular plants (**Supplementary Table 1**). We used this broad dataset to examine how pit membrane area and pit density vary among lineages and organs.

## 3 Results

### 3.1 Trait variation and physiological trait coordination

**Figure 2:**
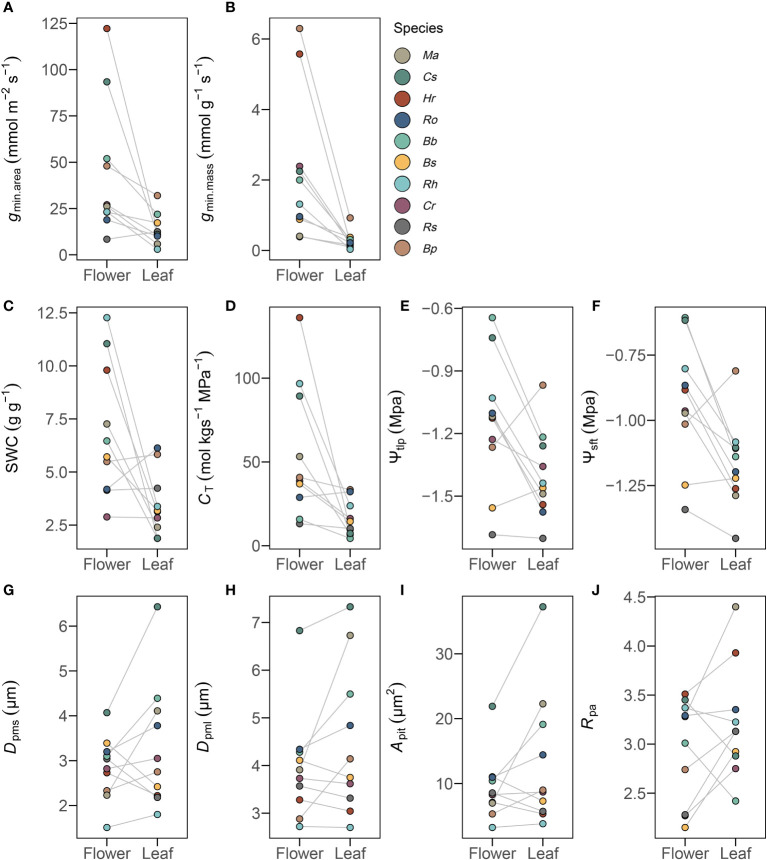
Traits differences of 10 selected traits for *g*_min_ **(A, B)**, pressure-volume curves **(C–F)**, and pit characteristics **(G–J)** in flowers and leaves. Mean values of listed traits from 10 species (n=10) were significantly different (*P*< 0.05) in flowers and leaves. See **Table 2** for definitions of abbreviations.

Minimum diffusive conductance (*g*_min_) was significantly higher in flowers than in leaves, whether it was normalized by dry mass (*t* = 5.48, *P*<0.001) or by projected area (*t* = 4.88, *P*<0.001) (**Figures 2A, B** and **Supplementary Table 2**). In addition, traits from pressure-volume curves were also significantly higher in flowers than in leaves (*P*< 0.01) (**Figures 2C–F** and **Supplementary Table 2**).

SEM images were used to examine intervessel pits in peduncles and petioles (**Figure 1B**). Compared to petioles, peduncles had significantly smaller pit membrane diameters *D*_pms_ (*t* = -2.36, *P*< 0.05) and *D*_pml_ (*t* = -2.86, *P*< 0.01) and smaller pit area *A*_pit_ (*t* = -3.09, *P*< 0.05), as well as differences in pit aperture shape *R*_pa_ (*t* = -2.16, *P*< 0.05) (**Figures 2G–J** and **Supplementary Table 2**). However, pit aperture diameters *D*_pas_ (*t* = -0.43, *P* > 0.05) and *D*_pal_ (*t* = -1.49, *P* > 0.05) and pit aperture area *A*_pa_ (*t* = -1.15, *P* > 0.05) were not significantly different between petioles and peduncles (**Supplementary Table 2**). Pit membrane shape *R*_pit_ (*t* = 0.41, *P* > 0.05) and pit density *D*_p_ (*t* = 2.02, *P* > 0.05) were similar in peduncles and petioles (**Supplementary Table 2**). Peduncles and petioles differed significantly in the size of the pit membranes despite having similar pit aperture sizes (**Figure 2** and **Supplementary Table 2**).

**Figure 3:**
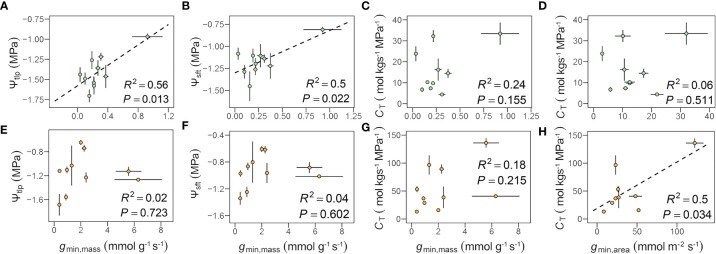
Relationships among minimum diffusive conductance (gmin) and traits from pressure-volume curves **(A–H**, see **Table 2** for definitions of abbreviations.). Each point represents the mean value in peduncles and petiole, respectively. The green circles represent leaf, the orange circles represent flower, and error bars represent standard error (n = 5 individual plants).

In leaves, *g*_min,mass_ was positively correlated with Ψ_tlp_ (*R* ^2 ^= 0.56, *P* = 0.013) and Ψ_sft_ (*R* ^2 ^= 0.50, *P* = 0.022), which remained significant after accounting for shared evolutionary history (**Figures 3A, B** and **Table 3**). No similar relationships between *g*_min,area_ or *g*_min,mass_ and *C*_T_ in leaves (**Figures 3C, D**) or *g*_min,mass_ and Ψ_tlp_ or Ψ_sft_ in flowers (**Figures 3E, F**) were found. *g*_min,area_ was positively correlated with *C*_T_ in flowers (*R* ^2 ^= 0.50, *P* = 0.034, **Figure 3H**). The relationships between *C*_T_ and both *g*_min,mass_ and *g*_min,area_ were significant after accounting for shared evolutionary history (**Table 3**).

**Table 3:** Phylogenetic independent contrast (PIC) results for paired traits of the 10 species studied, showing the PICs calculations between traits in flower and leaf.

		R^2^	
		Flower	Leaf
*D*_p_	*D*_pml_	0.93**	0.90**
	*D*_pms_	0.89**	0.89**
	*A*_pit_	0.95**	0.86**
	*D*_pal_	0.84**	0.84**
	*D*_pas_	0.93**	0.85**
	*A*_pa_	0.84**	0.81**
*A*_pit_	*A*_pa_	0.92**	0.92**
	*D*_h_	0.26	0.56*
*K*_th_	*D*_pas_	0.30	0.71**
	*D*_pal_	0.19	0.79**
	*A*_pit_	0.19	0.87**
*T*_w_	*A*_pit_	0.001	0.43
*D*_pal_	*T*_w_	0.03	0.49*
	*D*_h_	0.34	0.63*
	*D*_s_	0.002	0.39
*R*_pa_	*D*_h_	0.17	0.04
	*K*_th_	0.26	0.25
	SWC	0.37	0.22
	Ψ_sft_	0.23	0.22
	Ψ_tlp_	0.20	0.38
	*C*_T_	0.26	**0.02**
	FT/LT	0.31	0.72**
*R*_pit_	*D*_v_	0.02	0.66**
*g*_min,mass_	Ψ_sft_	0.001	0.50*
	Ψ_tlp_	0.01	0.45*
	*C*_T_	0.61*	0.72
*g*_min,area_	*C*_T_	0.54*	0.17
VF	*A*_pit_	0.10	0.09
	*A*_pa_	0.19	0.09

The numbers represent the correlation coefficients, *: significant correlation (P < 0.05), **: significant correlation (P < 0.01), ***: significant correlation (P < 0.001).

### 3.2 Trade off among intervessel pit traits

**Figure 4:**
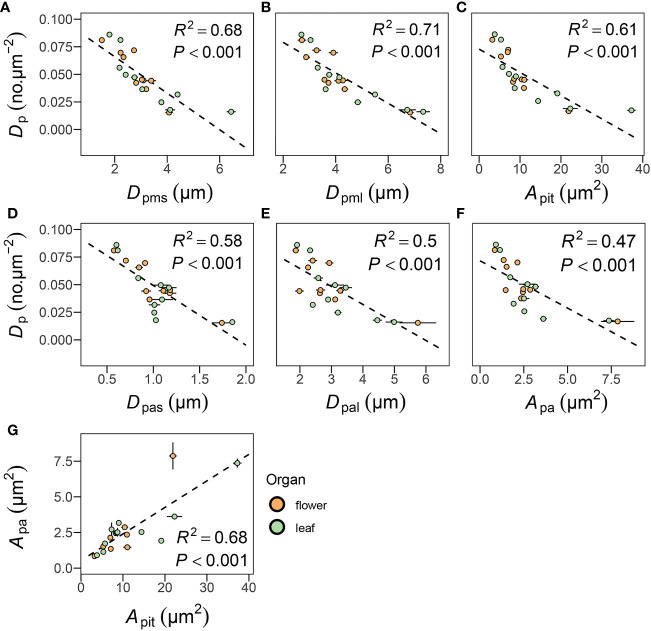
Relationships between pit density (*D*_p_) and pit membrane **(A–C)** and pit aperture traits **(D–F)**, and relationships between pit membrane area (*A*_pa_) with pit aperture area (*A*_pit_) **(G)**. Each point represents the mean value in peduncles and petiole, respectively. The green circles represent leaf, the orange circles represent flower, and error bars represent standard error (n = 3-5 individual plants).

Pit density *D*_p_ was negatively correlated with pit membrane diameters *D*_pms_ (*R* ^2 ^= 0.68, *P*< 0.001) and *D*_pml_ (*R* ^2 ^= 0.71, *P*< 0.001) and with pit area *A*_pit_ (*R* ^2 ^= 0.61, *P*< 0.001), as well as with pit aperture diameters *D*_pas_ (*R* ^2 ^= 0.58, *P*< 0.001) and *D*_pal_ (*R* ^2 ^= 0.5, *P*< 0.001) and pit aperture area *A*_pa_ (*R* ^2 ^= 0.47, *P*< 0.001) (**Figure 4**). These correlations remained significant after accounting for shared evolutionary history (all *P*< 0.05, **Table 3**). Comparing these data to previously published data from stems and leaves of a broad sampling of angiosperms, gymnosperms, and ferns (**Supplementary Table 1**) showed that the negative correlation between *D*_p_ and *A*_pit_ was common (**Supplementary Figure 1**), with gymnosperms exhibiting larger pits that occur at lower density (**Table 4** and **Supplementary Figure 1**). *A*_pit_ and *D*_p_ in flowers and leaves measured here were within the range reported previously for angiosperms and differed significantly from only gymnosperms (**Table 4**).

**Table 4:** Variation in *D*_p_ and *A*_pit_ among Fern, Gymnosperm and original data.

	*A*_pit_ (µm^2^)	*D*_p_ (no.µm^-2^)
Fern	11.23 ± 3.65**a**	0.073 ± 0.0179**a**
Gymnosperm	174.74 ± 25.09**b**	0.004 ± 0.0008**b**
Leaf	13.29 ± 1.84**a**	0.045 ± 0.0043**a**
Flower	9.38 ± 0.90**a**	0.052 ± 0.0035**a**

Different lower-case letters following the values indicate significant differences between groups (*P *< 0.05, LSD’s *post hoc* test, one-way ANOVA, values are means ± SE). Data for angiosperms, gymnosperms, and ferns were collected from published references (**Table S1**).

### 3.3 Relationships among pit traits and hydraulic traits

**Figure 5:**
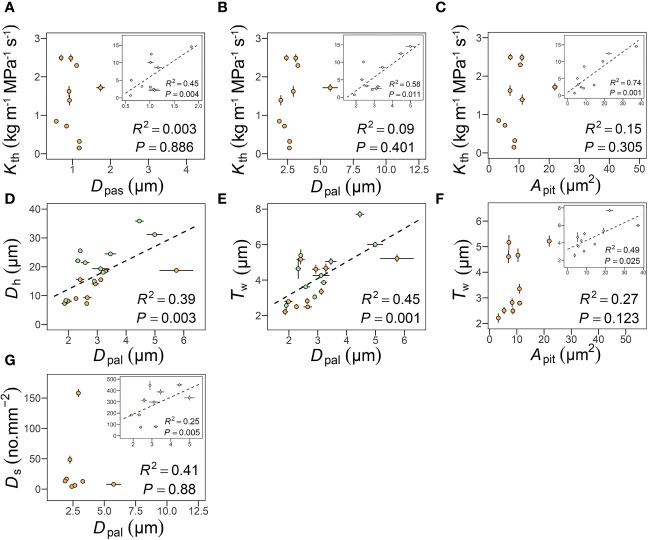
Relationships among pit apertures traits and theoretical hydraulic conductance (*K*_th_) **(A, B)**, pit membrane area and *K*_th_ **(C)** and double vessel wall thickness (*T*_w_) **(F)**, pit apertures traits and vessel diameter **(D)**, and double vessel wall thickness **(E)**, and stomatal density **(G)**. Each point represents the mean value in peduncles and petiole, respectively. The green circles represent leaf, the orange circles represent flower, and error bars represent standard error (n = 3-5 individual plants).

In petioles, like in other species (Lens et al., 2011; Mrad et al., 2018), *K*_th_ was positively correlated with *D*_pas_ (*R* ^2 ^= 0.45, *P* = 0.004), *D*_pal_ (*R* ^2 ^= 0.58, *P* = 0.011), and *A*_pit_ (*R* ^2 ^= 0.74, *P* = 0.001), but in peduncles these relationships were not significant, mainly because of the relatively constant *D*_pas_ and *D*_pal_ (**Figures 5A-C**). These correlations were statistically similar after accounting for shared evolutionary history (*P*< 0.05, **Table 3**). *D*_pal_ was positively correlated with *D*_h_ (*R* ^2 ^= 0.39, *P* = 0.003) and with *T*_w_ (*R* ^2 ^= 0.45, *P* = 0.001) in both petioles and peduncles (**Figures 5D, E**). These correlations remained significant only in leaves after accounting for shared evolutionary history (*P*< 0.05, **Table 3**). The positive relationships between *D*_pal_ and *D*_s_ (*R* ^2 ^= 0.25, *P* = 0.005) (**Figure 5G**), *A*_pit_ and *T*_w_ (*R* ^2 ^= 0.49, *P* = 0.025) (**Figure 5F**) became non-significant in leaves after accounting for shared evolutionary history (**Table 3**).

**Figure 6:**
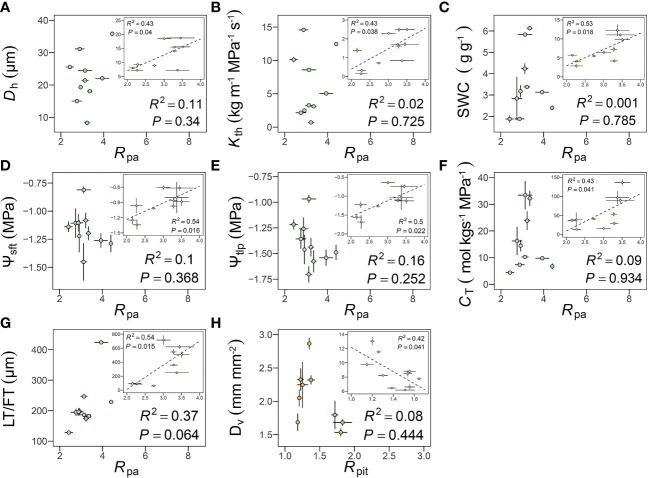
Relationships among pit membrane shape (*R*_pit_), pit apertures shape (*R*_pa_) and hydraulic traits. **(A–H**, see **Table 2** for definitions of abbreviations). Each point represents the mean value in peduncles and petiole, respectively. The green circles represent leaf, the orange circles represent flower, and error bars represent standard error (n = 3-5 individual plants).

In peduncles, *R*_pa_ was positively correlated with *D*_h_ (*R* ^2 ^= 0.43, *P* = 0.04), *K*_th_ (*R* ^2 ^= 0.43, *P* = 0.038), SWC (*R* ^2 ^= 0.53, *P* = 0.018), Ψ_sft_ (*R* ^2 ^= 0.54, *P* = 0.016), Ψ_tlp_ (*R* ^2 ^= 0.50, *P* = 0.022), *C*_T_ (*R* ^2 ^= 0.43, *P* = 0.041), FT (*R* ^2 ^= 0.54, *P* = 0.015) (**Figures 6A-G**). However, none of these correlations remained significant after accounting for shared evolutionary history (**Table 3**). *R*_pa_ was unrelated to any of these hydraulic traits in petioles, but *R*_pa_ was positively correlated with leaf thickness after accounting for shared evolutionary history (*R* ^2 ^= 0.72, *P*< 0.01) (**Table 3**). Only *R*_pit_ was negatively correlated with *D*_v_ (*R* ^2 ^= 0.42, *P* = 0.041) (**Figure 6H**), even after accounting for shared evolutionary history (*R* ^2 ^= 0.72, *P*< 0.01) (**Table 3**).

### 3.4 Phylogenetic independent contrast correlations of all paired traits between flowers and leaves

**Figure 7:**
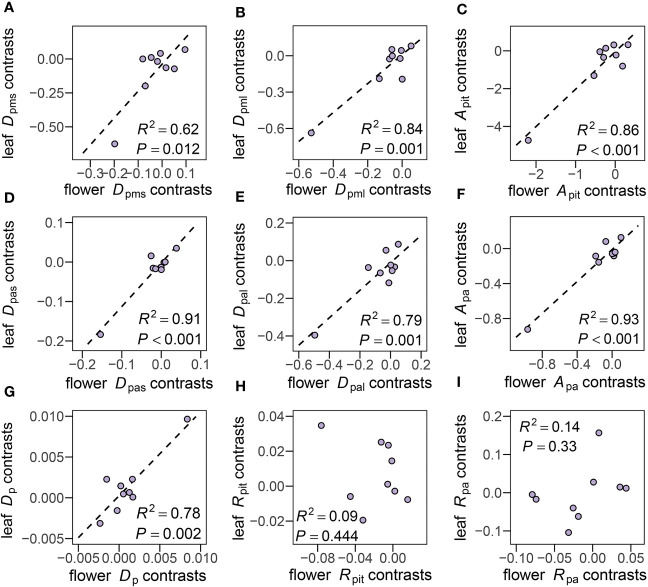
Phylogenetic independent contrast (PIC) correlations for pit traits. **(A–I**, see **Table 2** for definitions of abbreviations) of the 10 species studied, showing the PICs calculated for pit traits between flowers and leaves. Correlation coefficients and P values are shown for statistically significant correlations based on Pearson's product-moment correlation.

Phylogenetic independent contrast correlations (PIC) were made between all 24 traits measured in both flowers and leaves. Positive correlations of *D*_pms_ (*R* ^2 ^= 0.62, *P* = 0.012), *D*_pml_ (*R* ^2 ^= 0.84, *P* = 0.001), *A*_pit_ (*R* ^2 ^= 0.86, *P*< 0.001), *D*_pas_ (*R* ^2 ^= 0.91, *P*< 0.001), *D*_pal_ (*R* ^2 ^= 0.79, *P* = 0.001), *A*_pa_ (*R* ^2 ^= 0.93, *P*< 0.001), *D*_p_ (*R* ^2 ^= 0.78, *P* = 0.002) between flowers and leaves were found (**Figures 7A–G**), and species with larger pit membranes and pit apertures in petioles also had larger pit membranes and pit apertures in peduncles (**Supplementary Figure 2**). Interestingly, pit membrane (*R* ^2 ^= 0.09, *P* = 0.444) and pit aperture shape (*R* ^2 ^= 0.14, *P* = 0.33) showed non-significant relationships after accounting for shared evolutionary history (**Figures 7H, I**).

There were positive correlations in traits between organs for *S*_s_ (*R* ^2 ^= 0.50, *P* = 0.048), *D*_v_ (*R* ^2 ^= 0.41, *P* = 0.047), *T*_w_ (*R* ^2 ^= 0.49, *P* = 0.024), and *g*_min,mass_ (*R* ^2 ^= 0.46, *P* = 0.031) (**Supplementary Figure 3**), which became non-significant after accounting for shared evolutionary history (**Table 5**). Meanwhile some traits were not correlated among organs and remained uncorrelated even after accounting for shared evolutionary history: *g*_min,area_ (*R* ^2 ^= 0.13, *P* = 0.336), SWC (*R* ^2 ^= 0.07, *P* = 477), *K*_th_ (*R* ^2 ^= 0.06, *P* = 0. 523), Ψ_sft_ (*R* ^2 ^= 0.44, *P* = 0. 051), C_T_ (*R* ^2 ^= 0.02, *P* = 0.721), *D*_s_ (*R* ^2 ^= 0.13, *P* = 0.336), and LT/FT (*R* ^2 ^= 0.04, *P* = 0.626) (**Table 5**). Among physiological traits, only Ψ_tlp_ exhibited correlated evolution among flowers and leaves (*R* ^2^ = 0.54, *P* = 0.024) (**Table 5**).

**Table 5:** Phylogenetic independent contrast (PIC) correlations of all traits between flowers and leaves for the 10 species studied.

Trait	p-value	R^2^
*D*_pms_	<0.001	0.62**
*D*_pml_	0.001	0.84**
*A*_pit_	<0.001	0.86**
*D*_pas_	<0.001	0.91**
*D*_pal_	0.001	0.79**
*A*_pa_	<0.001	0.93**
*R*_pit_	0.444	0.09
*R*_pa_	0.330	0.14
*D*_p_	0.002	0.78**
*g*_min,area_	0.336	0.13
*g*_min,mass_	0.203	0.22
SWC	0.477	0.07
*K*_th_	0.523	0.06
Ψ_sft_	0.051	0.44
Ψ_tlp_	0.024	0.54**
*C*_T_	0.721	0.02
*S*_s_	0.201	0.22
*D*_s_	0.336	0.13
*D*_v_	0.100	0.34
LT/FT	0.626	0.04
*D*_h_	0.058	0.42
*T*_w_	0.096	0.35
VF	0.101	0.34

The numbers represent the correlation coefficients, *, significant correlation (P < 0.05); **, significant correlation (P < 0.01); ***, significant correlation (P < 0.001).

### 3.5 Principal component analysis

**Figure 8:**
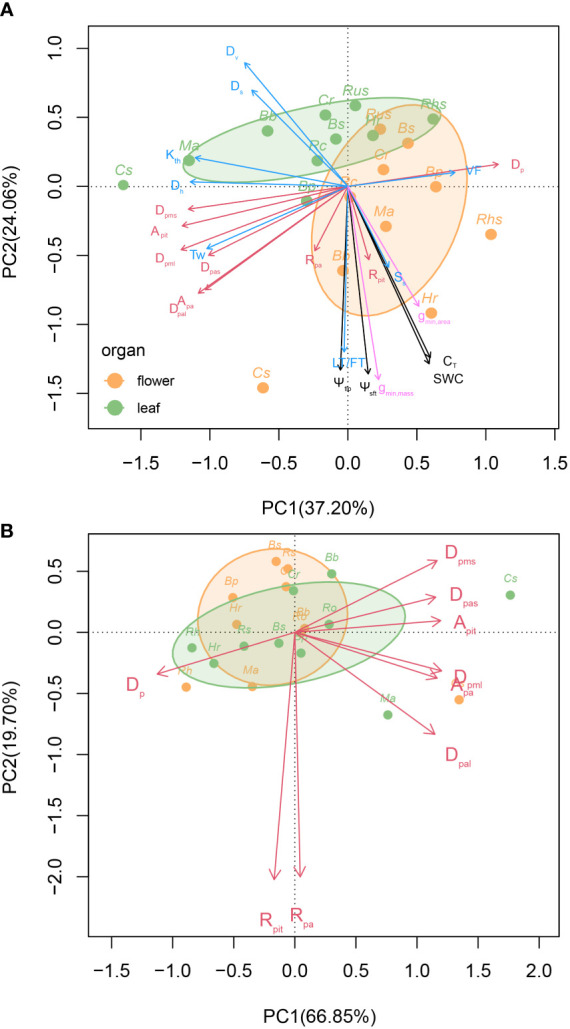
Principal component analysis (PCA) of all 24 traits on the first two principal component axes in flowers and leaves. The shaded regions indicate the total volume of trait space occupied by leaves (green) and flowers (orange). The red lines represent scanning electron microscopy anatomical traits, the black lines represent Pressure–volume parameters, the pink lines represent minimum diffusive conductance, and the blue lines represent light microscopy anatomical traits **(A)**. Principal component analysis of 9 pit traits on the first two principal component axes in flowers and leaves. The green circles represent leaf, the orange circles represent flower, and the shaded regions indicate the total volume of trait space occupied by leaves (green) and flowers (orange) **(B)**. See **Table 2** for definitions of abbreviations.

The principal components analysis using all 24 traits revealed that the first two principal components explained 37.20% and 24.06% of the total variation, respectively. The first PC was driven by *D*_h_, *K*_th_, and some pit characters, including *D*_p_, *D*_pms_, and *A*_pit_. The second PC was largely driven by pressure-volume parameters, including SWC, C_T_, Ψ_tlp_, Ψ_sft_, as well as anatomical traits, including *D*_v_, *D*_s_, and LT. Flowers and leaves largely differed in the regions of trait space they occupied (**Figure 8A**).

Using only the pit traits in a principal components analysis revealed that the first two principal components explained 86.55% of the total variation among species and organs. The first PC (66.85%) was driven primarily by *D*_p_ versus all of the other pit traits except *R_pit_* and *R_pa_*. The second PC (19.70%) was driven primarily by *R*_pit_ and *R*_pa_. There was a high level of overlap among flowers and leaves in the regions of pit trait space they occupied (**Figure 8B**).

## 4 Discussion

Our results revealed that despite large differences in *g*_min_ and pressure-volume traits between leaves and flowers, there were relatively small differences in pit traits between leaf petioles and flower peduncles (**Figure 8**). While flowers have higher *g*_min_ that is associated with higher hydraulic capacitance and higher turgor loss points than leaves (**Figures 2**, **3** and **Supplementary Table 2**), these differences in tissue water relations seem to be independent of differences in intervessel pit traits between organs. Thus, flowers rely on a cheap hydrostatic skeleton maintained by turgor pressure rather than a rigid, carbon-based skeleton (Roddy et al., 2019). High hydraulic capacitance of flowers prevents water potential declines that lead to xylem embolism and may have shielded selection from driving large divergences in intervessel pit traits between leaves and flowers.

### 4.1 Traits regulating water balance in flowers

In order for terminal organs to avoid desiccation, water loss must equal water supply, at least over diel timescales. In leaves, which maintain relatively high transpiration rates, the need to maintain water balance has resulted in coordinated evolution of key anatomical traits that influence both liquid water supply and water vapor loss, particularly leaf vein density (*D*_v_) and stomatal density and size (Sack et al., 2003; Brodribb et al., 2013; Simonin and Roddy, 2018; Zhang et al., 2018). While similar coordination between veins and stomata has been observed in flowers (Zhang et al., 2018), flowers often have few or no stomata, meaning that other traits, such as *g*_min_, may be more important to regulating water balance (Roddy et al., 2016; Roddy, 2019). Furthermore, in both flowers and leaves, higher water contents and hydraulic capacitance can buffer water potential declines and lengthen the time required to reach steady state transpiration when water supply equals water loss (Simonin et al., 2013; Roddy et al., 2018; Roddy et al., 2019). Because flowers can have higher *g*_min_ than leaves (**Figure 2**), we predicted that there may be coordination between *g*_min_ and hydraulic capacitance, which would indicate that higher hydraulic capacitance can compensate for higher *g*_min_ in flowers. Across organs, *g*_min_ and hydraulic capacitance were correlated even after accounting for shared evolutionary history (**Figure 3** and **Table 3**), with flowers having both higher *g*_min_ and higher capacitance than leaves (**Figure 2**). These patterns suggest that multiple traits and hydraulic strategies may be employed to maintain water balance among leaves and flowers.

In the absence of high hydraulic capacitance to buffer water potential declines, high *g*_min_ may cause water potentials to decline enough to initiate xylem embolism in flowers before leaves (Bourbia et al., 2020). If this were the case, we would predict that to prevent embolism in flowers, intervessel pit traits may have experienced selection to reduce embolism vulnerability. However, there were overall relatively small differences in intervessel pit traits between petioles and peduncles (**Figure 2**). One possible explanation is that intervessel pit traits may be shielded from selection by high hydraulic capacitance in flowers that allows *g*_min_ to be high without causing water potential declines and embolism spread. Because intervessel pit traits also influence hydraulic conductance (Choat et al., 2005; Ellmore et al., 2006; Hacke et al., 2006; Lens et al., 2011; Jacobsen et al., 2016), differences in intervessel pit traits between leaves and flowers may be due to divergent selection on hydraulic conductance among leaves and flowers. However, while flowers generally have relatively low hydraulic conductance, they are not necessarily outside the range of hydraulic conductance of leaves (Roddy et al., 2016), further suggesting that the strength of selection due to hydraulic efficiency acting on pit traits may be relatively weak.

### 4.2 Similar coordination of intervessel pit traits in leaves and flowers

In some cases, leaves and flowers exhibited similar coordination between intervessel pit traits despite the large morphological, anatomical, and physiological differences between these organs. We found similar coordination between *A*_pit_ and *A*_pa_ in both leaves and flowers (**Figure 4**), with larger *A*_pit_ being associated with higher theoretical hydraulic conductivity *K*_th_ in leaves (**Figure 5**). It is worth nothing that *K*_th_ incorporates only vessel traits and not intervessel pit traits, so coordination between *K*_th_ and *A*_pit_ and *A*_pa_ suggests that variation in vessel size and density are linked to intervessel pit variation. A broad sampling of vascular plants (**Supplementary Figure 1**) revealed strong coordination between *A*_pit_ and pit density (*D*_p_), due largely to packing constraints similar to those elucidated for stomata on the leaf surface and mesophyll cells inside the leaf (Franks and Beerling, 2009; Théroux-Rancourt et al., 2021; Borsuk et al., 2022; Jiang et al., 2023). Compared to other vascular plants, angiosperm *A*_pit_ and *D*_p_ were closer to the theoretical packing limit, which may be important to increasing hydraulic efficiency of angiosperm xylem regardless of other xylem traits. Coordination between *A*_pit_ and *D*_p_ was also found among flower peduncles and leaf petioles, similar to other angiosperms, regardless of the fact that previously published data were taken from both stems and leaves (**Supplementary Figure 1** and **Supplementary Table 1**). Previous studies have shown that larger *A*_pit_ is associated with larger *A*_pa_, leading to higher hydraulic conductivity (Orians et al., 2004; Wheeler et al., 2005; Lens et al., 2011; Johnson et al., 2016). Similarly, we found that *A*_pit_ was positively linked to *K*_th_ in leaves but not in flowers (noted that *A*_pit_ was positively correlated with *D*_h_ but not VF in leaves, **Table 3**), suggesting that coordination in pit traits and vessel dimensions and packing were decoupled in flowers (**Figure 5**). Thus, despite obeying similar biophysical packing principles as vegetative organs–i.e. stomatal and vein densities (Zhang et al., 2018)–flowers can deviate in other traits that also influence hydraulic performance.

### 4.3 Special pit traits and correlations in flowers

From the hydraulic efficiency perspective, plants that have larger diameter vessels will have higher hydraulic efficiency (Hargrave et al., 1994; Christenhusz et al., 2011; Liu et al., 2019), while larger conduit diameter, larger pit membrane area, and larger pit aperture area, will be expected to decrease hydraulic safety (Pittermann et al., 2010; Lens et al., 2011; Brodersen et al., 2014; Jacobsen et al., 2016). Conduit wall thickness is thought increase hydraulic safety (Hacke et al., 2001; Brodribb and Holbrook, 2005). In our results, both pit membrane and pit aperture traits were correlated with *K*_th_ and *D*_h_ in leaves, no such correlations were found in flowers (**Table 3**). These results may indicate that leaves increase hydraulic efficiency with larger vessels and *A*_pit_, but they may increase hydraulic safety through thicker vessel walls and more elliptical pits (**Table 3**). On the other hand, high hydraulic capacitance of flowers prevents water potential declines may relax the selective pressure of intervessel pit traits for hydraulic efficiency and safety. In general, elliptically shaped pit apertures are associated with greater embolism resistance, with more cavitation-resistant species exhibiting narrower and more elliptical pit apertures (Lens et al., 2011; Scholz et al., 2013; Jacobsen et al., 2016). Consequently, angiosperms adapted to dry environments might have smaller conduit diameters and thicker, denser, smaller, and more elliptical pit apertures (Wheeler et al., 2005; Hacke et al., 2007; Jansen et al., 2009; Lens et al., 2011; Scholz et al., 2013). While intervessel pit traits might influence both hydraulic safety and efficiency, none of the pit traits were correlated with hydraulic traits in flowers (**Table 3**). It is highly likely, therefore, that traits exhibiting greater differences between leaves and flowers (e.g. pressure-volume traits) may be more important to flower water balance than intervessel pit traits.

Flowers have been shown to exhibit high diversity in hydraulic traits with higher water content and higher hydraulic capacitance than leaves (Roddy et al., 2019). We found similar patterns in our data, with flowers exhibiting greater variation in *g*_min_ and pressure-volume traits than leaves (**Figure 2**). However, flowers exhibited less variation in pit traits than leaves (**Figure 2**), although there were some clear differences in intervessel pit traits between leaves and flowers. This was further validated by the PCA results (**Figure 8**), in which *R*_pit_ and *R*_pa_ loaded on the same axis as the pressure-volume traits, in contrast to all other intervessel pit traits, which were orthogonal to other hydraulic traits except *D*_h_ and *K*_th_. Further corroborating the role of *R*_pit_ and *R*_pa_ in causing the divergence in hydraulic strategies between leaves and flowers, *R*_pit_ and *R*_pa_ exhibited no correlated evolution between leaves and flowers, in contrast to all other intervessel pit traits (**Figure 7**). Taken together, these results suggest that while most of the hydraulic differences between leaves and flowers is due to stomatal and vein anatomy and pressure-volume traits, differences in pit and pit aperture shape may also signify important differences.

## 5 Conclusions

The water dynamics of flowers are critical to successful reproduction and population viability, yet remarkably little is known about the hydraulic strategies of flowers and their mechanisms of maintaining water balance. Limiting water loss, storing large amounts of water, and building xylem safe from embolism are all ways of avoiding the detrimental effects of water limitation. Here we show that, compared to leaves, flowers are leakier and exhibit relatively few differences in intervessel pit traits that influence embolism vulnerability. Instead, flowers primarily use high water contents to prevent water potential declines. This drought-avoidant strategy employed by flowers may have protected their xylem from selection for greater differences from leaves. Furthermore, by quantifying a broad suite of anatomical and physiological traits among leaves and flowers, we show that with the exception of pit and pit aperture shape, intervessel pit traits are largely orthogonal to stomatal and vein traits and pressure-volume traits. These results highlight the many dimensions in which flowers have diverged from leaves under different functional demands and suggest that high water content and hydraulic capacitance are the primary traits that protect flowers from experiencing low water potentials that can cause failure in the hydraulic system.

## Data availability statement

The original contributions presented in the study are included in the article/**Supplementary Material**. Further inquiries can be directed to the corresponding author.

## Author contributions

G-FJ conceived the ideas and designed the study. Y-DA, AR and G-FJ collected the data. Y-DA, T-HZ and G-FJ analyzed the data. G-FJ and Y-DA wrote the first manuscript, AR helped to improve final manuscript; and all authors reviewed each draft before giving approval for submission of the final version. All authors contributed to the article and approved the submitted version.
