# Supplementary material for: Hydraulic differences between flowers and leaves are driven primarily by pressure-volume traits and water loss
Source: Front Plant Sci. 2023 May 31;14:1130724. doi: 10.3389/fpls.2023.1130724 (PMC10264769; doi:10.3389/fpls.2023.1130724)
Supplement: Supplementary file 1 [file DataSheet_1.pdf]

Supplementary Material

Hydraulic differences between flowers and leaves is driven primarily by pressure-volume traits and water loss

Yi-Dong An[1], Adam B. Roddy[2], Tian-Hao Zhang[1], Guo-Feng Jiang[1*]

*** Correspondence:** Corresponding Author: gfjiang@gxu.edu.cn (G.-F.J.)

1 Supplementary Figures and Tables

1.1 Supplementary Figures

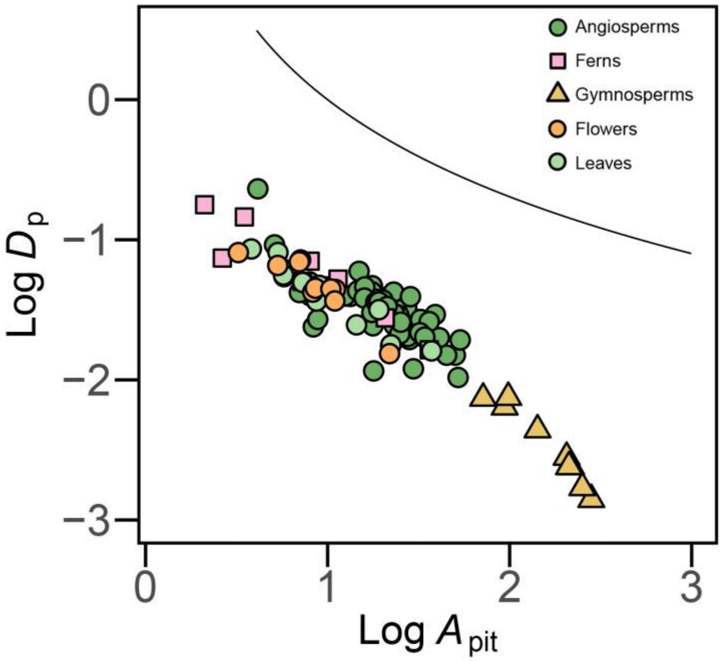

Supplementary Figure 1. The relationship between intervessel pit membrane area (A_{pit}) and pit density (D_p) among different plant groups. Each point is an individual species. The curved line is the estimated upper theoretical limit to pit density at a given pit size area. The dark green circles represent angiosperms, the pink squares represent ferns, the yellow triangles represent gymnosperms, the orange circles and the light green circles represent original data of flowers and leaves from present study, respectively. Additional information for pit traits from published papers are included in Supplementary Table 1.

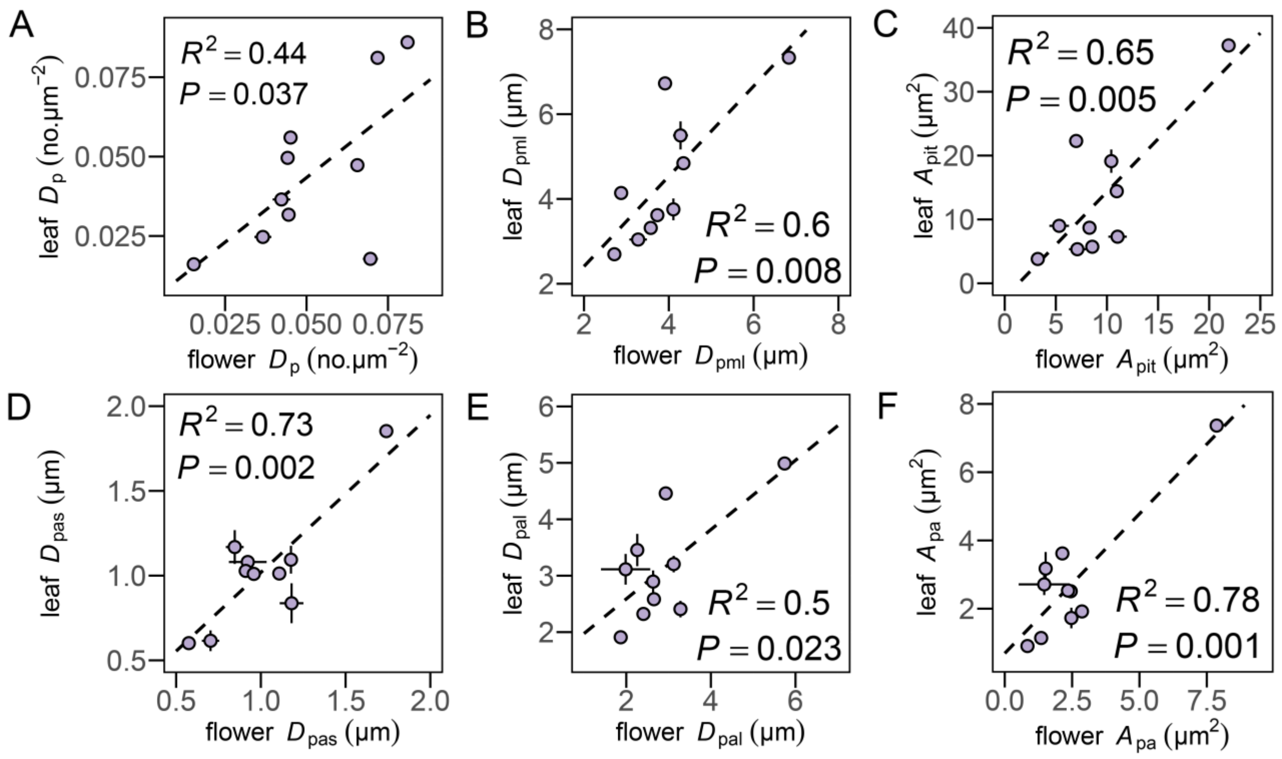

Supplementary Figure 2. Pairwise trait correlations of pit traits between flowers and leaves. Each point represents the mean value, error bars represent standard error (n = 3 individual plants, at least 50 pits measured per plant). See Table 2 for definitions of abbreviations.

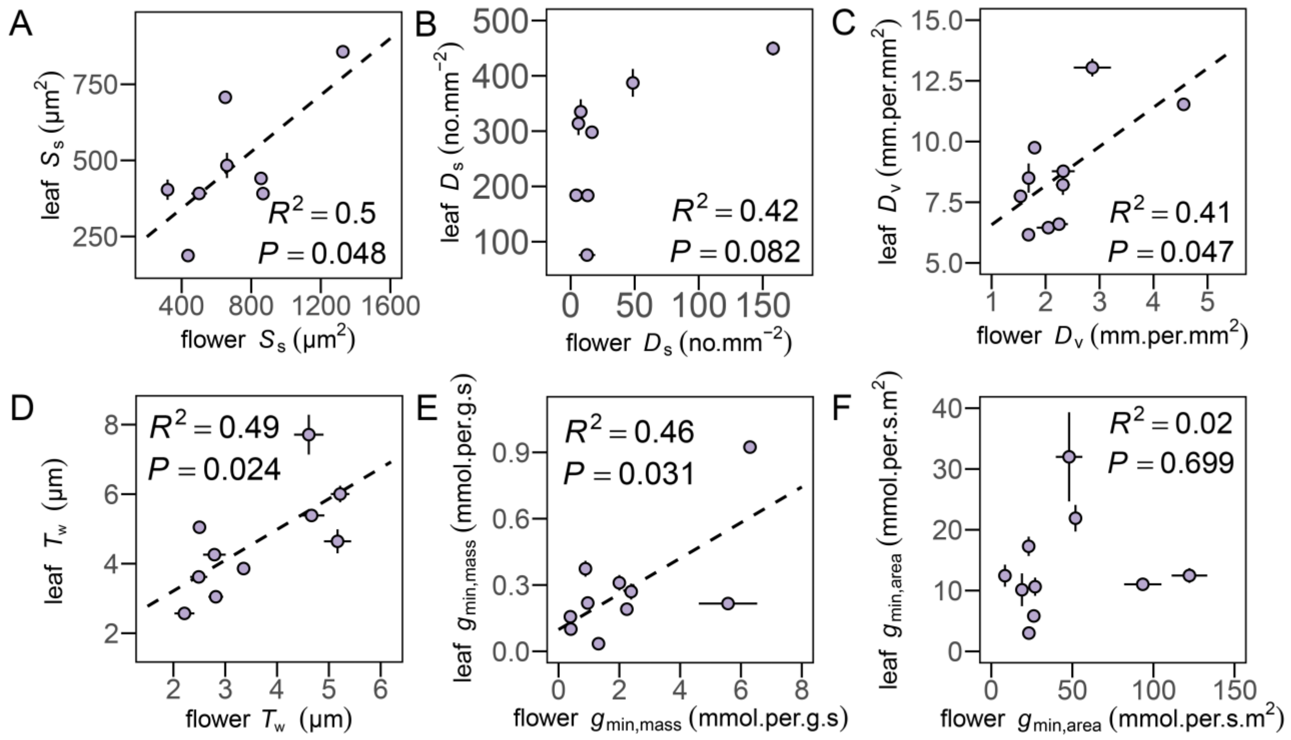

Supplementary Figure 3. Pairwise trait correlations of hydraulic traits. Each point represents the mean value, error bars represent standard error (n = 3-5 individual plants). See Table 2 for definitions of abbreviations.

1.2 Supplementary Tables

Supplementary Table 1. Additional pit traits information for Supplementary Figure 1.

**Group**	**Genus**	**Species**	**Organ**	**A_{pit} (μm²)**	**D_p (no. μm⁻²)**	**Literature**
Angiosperm	Ficus	*Ficus altissima*	Leaf	41.10	0.0200	(Li et al., 2019)
Angiosperm	Ficus	*Ficus benjamina*	Leaf	50.30	0.0150	
Angiosperm	Ficus	*Ficus concinna*	Leaf	28.70	0.0280	
Angiosperm	Ficus	*Ficus curtipes*	Leaf	45.10	0.0150	
Angiosperm	Ficus	*Ficus virens*	Leaf	29.80	0.0210	
Angiosperm	Ficus	*Ficus callosa*	Leaf	17.70	0.0350	
Angiosperm	Ficus	*Ficus esquiroliana*	Leaf	27.20	0.0330	
Angiosperm	Ficus	*Ficus fistulosa*	Leaf	21.10	0.0390	
Angiosperm	Ficus	*Ficus hispida*	Leaf	17.60	0.0470	
Angiosperm	Ficus	*Ficus semicordata*	Leaf	32.40	0.0270	
Angiosperm	Acer	*Acer pseudoplatanus*	Stem	39.03	0.0292	(Kotowska

Angiosperm	Acer	*Acer campestre*	Leaf	17.75	0.0243	et al., 2020)
Angiosperm	Acer	*Acer campestre*	Stem	26.60	0.0220	(Wu et al., 2020)
Angiosperm	Acer	*Acer pseudoplatanus*	Stem	29.20	0.0210	
Angiosperm	Corylus	*Corylus avellana*	Stem	23.90	0.0320	
Angiosperm	Fraxinus	*Fraxinus americana*	Stem	12.50	0.0396	(Choat et al., 2006)
Angiosperm	Ulmus	*Ulmus americana*	Stem	53.50	0.0193	
Angiosperm	Betula	*Betula nigra*	Stem	4.14	0.2311	(Jansen et al., 2009)
Angiosperm	Quiina	*Quiina indigofera*	Stem	5.76	0.0546	(Jansen et al., 2004)
Angiosperm	Fraxinus	*Fraxinus americana*	Stem	14.89	0.0595	
Angiosperm	Guapira	*Guapira cuspidata*	Stem	23.06	0.0423	
Angiosperm	Anogeissus	*Anogeissus leiocarpus*	Stem	36.16	0.0261	(Jansen et al., 2008)
Angiosperm	Conostegia	*Conostegia xalapensis*	Stem	17.49	0.0426	
Angiosperm	Punica	*Punica protopunica*	Stem	33.74	0.0203	
Angiosperm	Adenostoma	*Adenostoma fasciculatum*	Stem	7.97	0.0500	(Jacobsen et al., 2016)
Angiosperm	Arctostaphylos	*Arctostaphylos glandulosa*	Stem	13.34	0.0394	
Angiosperm	Ceanothus	*Ceanothus leucodermis*	Stem	28.17	0.0194	
Angiosperm	Heteromeles	*Heteromeles arbutifolia*	Stem	12.47	0.0397	
Angiosperm	Quercus	*Quercus berberidifolia*	Stem	24.27	0.0200	
Angiosperm	Quercus	*Quercus wislizeni*	Stem	27.60	0.0204	
Angiosperm	Rhamnus	*Rhamnus crocea*	Stem	21.41	0.0294	
Angiosperm	Rhus	*Rhus ovata*	Stem	14.62	0.0432	
Angiosperm	Ribes	*Ribes petraeum*	Stem	18.26	0.0377	(Jansen et al., 2007)
Angiosperm	Rhamnus	*Rhamnus utilis*	Stem	29.60	0.0120	
Angiosperm	Zelkova	*Zelkova serrata*	Stem	32.12	0.0217	
Angiosperm	Cercocarpus	*Cercocarpus ledifolius*	Stem	52.12	0.0104	
Angiosperm	Cercocarpus	*Cercocarpus betuloides*	Stem	5.59	0.0625	
Angiosperm	Kerria	*Kerria japonica*	Stem	8.35	0.0239	
Angiosperm	Cotoneaster	*Cotoneaster integerrimus*	Stem	22.20	0.0174	
Angiosperm	Holodiscus	*Holodiscus dumosus*	Stem	8.04	0.0398	
Angiosperm	Lyonothamnus	*Lyonothamnus floribundus*	Stem	8.83	0.0270	
Angiosperm	Hemiptelea	*Hemiptelea davidii*	Stem	16.00	0.0461	
Angiosperm	Magnolia	*Magnolia grandiflora*	Leaf	20.14	0.0371	(Zhang et al., 2021)
Angiosperm	Magnolia	*Magnolia grandiflora*	Flower	28.52	0.0393	
Angiosperm	Magnolia	*Magnolia grandiflora*	Stem	17.91	0.0116	
Angiosperm	Prunus	*Prunus padus*	Stem	21.50	0.0330	(Scholz et

Angiosperm	Prunus	*Prunus cerasus*	Stem	20.10	0.0313	al., 2013)
Angiosperm	Prunus	*Prunus avium*	Stem	18.80	0.0346	
Angiosperm	Prunus	*Prunus persica*	Stem	19.50	0.0328	
Angiosperm	Prunus	*Prunus spinosa*	Stem	24.60	0.0289	
Angiosperm	Prunus	*Prunus mahaleb*	Stem	23.20	0.0246	
Angiosperm	Prunus	*Prunus domestica*	Stem	19.60	0.0352	
Angiosperm	Prunus	*Prunus armeniaca*	Stem	15.90	0.0384	
Angiosperm	Prunus	*Prunus dulcis*	Stem	20.50	0.0293	
Angiosperm	Prunus	*Prunus cerasifera*	Stem	18.40	0.0359	
Angiosperm	Lauraceae	*Laurus nobilis*	Stem	17.50	0.0303	(Jansen et al., 2011)
Angiosperm	Fagaceae	*Quercus ilex*	Stem	24.92	0.0213	
Angiosperm	Oleaceae	*Olea europaea*	Stem	5.11	0.0920	
Angiosperm	Acer	*Acer Campestre*		23.29	0.0296	(Klepsch et al., 2016)
Angiosperm	Acer	*Acer Monspessulanum*		25.23	0.0258	
Angiosperm	Acer	*Acer Platanatum*		18.54	0.0356	
Angiosperm	Acer	*Acer Pseudoplatanus*		34.18	0.0202	
Angiosperm	Acer	*Acer Sieboldianum*		19.15	0.0360	
Angiosperm	Acer	*Acer Tataricum.*		21.23	0.0334	
Angiosperm	Avicennia	*Avicennia marina*		7.00	0.0420	(Schmitz et al., 2007)
Fern	Ampelopteris	*Ampelopteris prolifera*	Stem	9.22	0.0469	(Laskar et al., 2020)
Fern	Dennstaedtia	*Dennstaedtia punctilobula*	Stem	20.74	0.0277	(Suissa and Friedman, 2021)
Fern	Parathelypteris	*Parathelypteris noveboracensis*	Stem	36.17	0.0164	
Fern	Marsilea	*Marsilea quadrifolia*	Stem	8.02	0.0703	
Fern	Neoblechnum	*Neoblechnum brasiliense*	Stem	7.25	0.0503	(Schneider, 2009)
Fern	Platycerium	*Platycerium bifurcatum*	Stem	11.41	0.0524	
Fern	Phlebodium	*Phlebodium aureum*	Stem	2.64	0.0740	(Pittermann et al., 2015)
Fern	Selaginella	*Selaginella pallescens*	Stem	2.11	0.1779	
Fern	Asplenium	*Asplenium nidus*	Root	3.49	0.1455	(Carlquist and Schneider, 2000)
Gymnosperm	Picea	*Picea orientalis*	Stem	281.10	0.0014	(Durmaz et al., 2016)
Gymnosperm	Ephedra	*Ephedra trifurca*	Stem	71.53	0.0074	(Jansen et al., 2014)
Gymnosperm	Abies		Stem	205.57	0.0028	(Wang et al., 2009)
Gymnosperm	Tsuga		Stem	217.51	0.0024	

Gymnosperm	Picea		Stem	250.71	0.0017	
Gymnosperm	Araucaria		Stem	93.91	0.0064	(Jacobsen, 2021)
Gymnosperm	Torreya		Stem	211.76	0.0024	
Gymnosperm	Agathis		Stem	142.29	0.0044	
Gymnosperm	Cycas		Stem	98.32	0.0075	

Supplementary Table 2. Differences in traits of minimum diffusive conductance (g_{min}), parameters from pressure-volume curves, and pits between flowers and leaves (*: P < 0.05, ** : P < 0.01, paired t-tests, values are means ± SE, n = 30 ~ 50).

Traits	Flower	Leaf	Test statistic
$g_{min,mass}$	2.25±0.34	0.28±0.04	**6.11****
$g_{min,area}$	44.23±5.22	13.67±1.38	**5.76****
SWC	7.11±0.53	3.50±0.28	**5.34****
Ψ_{sft}	-0.95±0.05	-1.17±0.04	**4.08****
Ψ_{tlp}	-1.17±0.06	-1.41±0.04	**3.71****
C_T	58.98±7.24	15.89±1.95	**5.64****
D_{pms}	2.84±0.13	3.31±0.25	**-2.36***
D_{pml}	3.96±0.21	4.50±0.28	**-2.86****
A_{pit}	9.38±0.90	13.29±1.84	**-3.09***
D_{pas}	1.01±0.06	1.03±0.06	-0.43
D_{pal}	2.89±0.20	3.13±0.18	-1.49
A_{pa}	2.53±0.36	2.76±0.33	-1.15
R_{pit}	1.44±0.05	1.41±0.03	0.41
R_{pa}	2.93±0.11	3.22±0.12	**-2.16***
D_p	0.052±0.0035	0.045±0.0043	2.02

Additional References and Notes:

Supplementary Table 1

Uncategorized References

Carlquist, S., and Schneider, E. (2000). SEM studies of vessels in ferns. *Aquatic Botany* 66**,** 1–8. doi: 10.1016/S0304-3770(99)00023-6.

Choat, B., Brodie, T.W., Cobb, A.R., Zwieniecki, M.A., and Holbrook, N.M. (2006). Direct measurements of intervessel pit membrane hydraulic resistance in two angiosperm tree species. *American Journal of Botany* 93(7)**,** 993-1000. doi: 10.3732/ajb.93.7.993.

Durmaz, S., Yildiz, Ü.C., Öztürk, M., and Serdar, B. (2016). Investigation of enzymatic effects on pit membranes using light and scanning electron microscopy. *Drewno* 59**,** 163-170. doi: 10.12841/wood.1644-3985.178.05.

Jacobsen, A. (2021). Diversity in conduit and pit structure among extant gymnosperm taxa. *American Journal of Botany* 108. doi: 10.1002/ajb2.1641.

Jacobsen, A.L., Tobin, M.F., Toschi, H.S., Percolla, M.I., and Pratt, R.B. (2016). Structural determinants of increased susceptibility to dehydration-induced cavitation in post-fire resprouting chaparral shrubs. *Plant, Cell & Environment* 39(11)**,** 2473-2485. doi: 10.1111/pce.12802.

Jansen, S., Baas, P., Gasson, P., Lens, F., and Smets, E. (2004). Variation in xylem structure from tropics to tundra: Evidence from vestured pits. *Proceedings of the National Academy of Sciences of the United States of America* 101**,** 8833-8837. doi: 10.1073/pnas.0402621101.

Jansen, S., Best, T., Elder, T., Schier, S., Pauline S, B., Vevon, A., et al. (2014). Pit membranes of Ephedra resemble gymnosperms more than angiosperms. *IAWA Journal* 35**,** 217-235. doi: 10.1163/22941932-00000062.

Jansen, S., Choat, B., and Pletsers, A. (2009). Morphological variation of intervessel pit membranes and implications to xylem function in angiosperms. *American journal of botany* 96**,** 409-419. doi: 10.3732/ajb.0800248.

Jansen, S., Gortan, E., Lens, F., Lo Gullo, M.A., Salleo, S., Scholz, A., et al. (2011). Do quantitative vessel and pit characters account for ion-mediated changes in the hydraulic conductance of angiosperm xylem? *New Phytologist* 189(1)**,** 218-228. doi: 10.1111/j.1469-8137.2010.03448.x.

Jansen, S., Pletsers, A., Rabaey, D., and Lens, F. (2008). Vestured pits: A diagnostic character in the secondary xylem of myrtales. *Journal of Tropical Forest Science* 20**,** 328-339.

Jansen, S., Sano, Y., Choat, B., Rabaey, D., Lens, F., and Dute, R. (2007). Pit membranes in tracheary elements of Rosaceae and related families: New records of tori and pseudotori. *American journal of botany* 94**,** 503-514. doi: 10.3732/ajb.94.4.503.

Klepsch, M., Schmitt, M., Knox, P., and Jansen, S. (2016). The chemical identity of intervessel pit membranes in Acer challenges hydrogel control of xylem hydraulic conductivity. *AoB Plants* 8**,** plw052. doi: 10.1093/aobpla/plw052.

Kotowska, M.M., Thom, R., Zhang, Y., Schenk, H.J., and Jansen, S. (2020). Within-tree variability and sample storage effects of bordered pit membranes in xylem of Acer pseudoplatanus. *Trees* 34(1)**,** 61-71. doi: 10.1007/s00468-019-01897-4.

Laskar, S., Ghoshal, U., and Sen, K. (2020). Vessel elements of two thelypteroid ferns-part I. *Botanical Studies* 61. doi: 10.1186/s40529-020-0281-y.

Li, S., Hao, G.Y., Niinemets, U., Harley, P.C., Wanke, S., Lens, F., et al. (2019). The effects of intervessel pit characteristics on xylem hydraulic efficiency and photosynthesis in hemiepiphytic and non-hemiepiphytic Ficus species. *Physiol Plant* 167(4)**,** 661-675. doi: 10.1111/ppl.12923.

Pittermann, J., Watkins, J.E., Cary, K.L., Schuettpelz, E., Brodersen, C., Smith, A.R., et al. (2015). "The Structure and Function of Xylem in Seed-Free Vascular Plants: An Evolutionary Perspective," in *Functional and Ecological Xylem Anatomy*.), 1-37.

Schmitz, N., Jansen, S., Verheyden, A., Kairo, J.G., Beeckman, H., and Koedam, N. (2007). Comparative Anatomy of Intervessel Pits in Two Mangrove Species Growing Along a Natural Salinity Gradient in Gazi Bay, Kenya. *Annals of botany* 100**,** 271-281. doi: 10.1093/aob/mcm103.

Schneider, S. (2009). Tracheary Elements in Ferns: New Techniques, Observations, and Concepts. *American Fern Journal***,** 199-211. doi: 10.1640/0002-8444(2007)97[199:TEIFNT]2.0.CO;2.

Scholz, A., Rabaey, D., Stein, A., Cochard, H., Smets, E., and Jansen, S. (2013). The evolution and function of vessel and pit characters with respect to cavitation resistance across 10 Prunus species. *Tree Physiology* 33(7)**,** 684-694. doi: 10.1093/treephys/tpt050.

Suissa, J.S., and Friedman, W.E. (2021). From cells to stems: the effects of primary vascular construction on drought-induced embolism in fern rhizomes. *New Phytologist* 232(6)**,** 2238-2253. doi: 10.1111/nph.17629.

Wang, Q., Zhang, Z.-L., Ding, H., Shao, W.-B., Li, C.-S., Wang, Y.-F., et al. (2009). The wood in the pits of terracotta figures and its architectural application. *Journal of Archaeological Science* 36(2)**,** 555-561. doi: 10.1016/j.jas.2008.10.016.

Wu, M., Ya, Z., Oya, T., Marcati, C., Pereira, L., and Jansen, S. (2020). Root xylem in three woody angiosperm species is not more vulnerable to embolism than stem xylem. *Plant and Soil* 450. doi: 10.1007/s11104-020-04525-0.

Zhang, F.P., Zhang, J.L., Brodribb, T.J., and Hu, H. (2021). Cavitation resistance of peduncle, petiole and stem is correlated with bordered pit dimensions in Magnolia grandiflora. *Plant Divers* 43(4)**,** 324-330. doi: 10.1016/j.pld.2020.11.007.
